# Supplementary material for: Development of the National Institute of Health Healing Experience of All Life Stressors Short Form (NIH-HEALS-SF)
Source: PLOS Ment Health. 2025 Mar 26;2(3):e0000276. doi: 10.1371/journal.pmen.0000276 (PMC12798453; doi:10.1371/journal.pmen.0000276)
Supplement: Attachment — Submitted filename: Response to Reviewers.docx [file pmen.0000276.s001.docx]

Dear editor and reviewers,

We appreciate your review and some suggestions have been justified below. We hope that our manuscript will be ready to be accepted into your prestigious journal after this round of review.

**Reviewer #1:** **Please review the sampling procedure and give rational based on scientific procedure to make better
need better interpretation of table and follow statistical norms for analysis
it it better to describe the information following the scientific procedure in methodology section.**

>>We thank the reviewer for raising these questions. We have expanded the methodology section of our manuscript to provide further clarify.

In summary, our study was based on a methodological approach for a scale reduction. Because the NIH-HEALS is a useful psychosocial-spiritual well-being tool validated among patients with serious and/or life-limiting illnesses, the sampling methodology included four independent groups (samples) of participants with varying degrees and types of chronic illness and stress. The entire set of participants were from four individual studies that by themselves utilized appropriate methods to enroll them. One of these studies (sample 4) also carried out randomization of treatments within the study, and another (sample 3) was based on hereditary factors. While strict sampling techniques may not have been used for participant selections within the included studies, importantly, our study included these cohorts who are representative of the target population to which the NIH-HEALS is applicable. This, in turn, maximized the extrapolation of our findings.

Changes: page 4, line 71, and lines 79-82.

**Reviewer #2: This manuscript addresses the development of the NIH-HEALS short form (NIH-HEALS-SF), a tool aimed at reducing patient burden while maintaining the robust psychometric properties of the original scale. The topic is highly relevant, as the optimization of psychosocial-spiritual assessment tools enhances their usability in both clinical and research settings. The methodology is appropriate for scale reduction, and the results are compelling, demonstrating that the shortened version retains strong reliability and validity. However, there are areas where the manuscript could benefit from further clarification and elaboration to strengthen its impact.**

**1. The use of LASSO regression for item reduction is a strength of the study. However, the manuscript would benefit from additional explanation of why this method was chosen over alternative item selection techniques, such as exploratory or confirmatory factor analysis. Highlighting the advantages of LASSO in this context would strengthen the rationale for the methodology. Why not using an EFA approach? Another piece of information is missing here is what statistical software/package is used here for conducting LASSO and the model fit indices were not reported here either.
I think more elaboration of this method and statistical output is needed for readers to better understand the process of item selection.**

>> Thank you for the recommendations. We have edited the statistical analysis selection to incorporate the missing information.

In short, exploratory (EFA) and confirmatory (CFA) factor analyses were indeed carried out during the validation phases of the NIH-HEALS. EFA was conducted to determine the underlying structures of the NIH-HEALS and the constructs that were identified by the resulting factors. CFA was used to validate the NIH-HEALS. These details were previously published and are described in detail elsewhere (reference 7); however, relevant results are included in the paragraph on the instrument.

Since the purpose of the current study was scale reduction and not validation, factor analyses were not used. LASSO regression was appropriate because we aimed to reduce the dimensionality of our data from NIH-HEALS and eliminate less important items. In addition, LASSO provided the advantages of a simpler tool that would be easier to use and interpret, works well with correlated items within the tool, and can reduce variance and overfitting of the data.

The last paragraph of the statistical analysis section includes the methodological details for model fit assessment, and the expected range and interpretation of findings. The last sentence in that paragraph describes the use of SAS v9.4 to conduct the analyses, and we have further added model specifications (proc glmselect, with selection=LASSO based on Schwarz Bayesian Criterion [choose=SBC]). In addition, the model’s item selection results are shown in Table 3, and specific SBC results are study and data-specific and not universal; thus, they may not be helpful in other settings as the output from such an analysis identifies the selections accordingly.

Changes: page 7, lines 122-126, and 130-132. Page 8, lines 150-151.

**2. It is unclear how the three factors (Connection, Reflection and Introspection, and Trust and Acceptance) guided the final selection of items. Were these factors weighted equally in the selection process? Including a brief discussion of the factor-specific contribution to the short form would provide greater transparency.**

>> Thank you for this suggestion. We have added more detail related to the factors in the statistical analysis section of the paper (pages 7-8, lines 137-139). The NIH-HEALS was validated among patients with serious and/or life-limiting illnesses and the resulting constructs were identified by the three factors of Connection, Reflection and Introspection, and Trust and Acceptance. It is important for the reduced scale of the short form NIH-HEALS-SF to also reflect these three factors. Thus, they were considered in the overall ranking and were also weighted to represent the full scale proportionally.

**3. The study mentions validation using data from three distinct samples. I think a discussion of whether the psychometric properties of the short form varied across subgroups (e.g., different clinical populations) would enhance the manuscript. Are there significant differences in the level of healing across different samples or populations.**

>> We thank the reviewer for this interesting question; however, we intended to keep the focus of the paper on the performance of the reduced scale NIH-HEALS-SF within each sample relative to the full scale NIH-HEALS 35-item. The addition of comparisons among the samples and their implications are beyond the scope of this paper. We have indeed reported the reliability for each sample and discussed sample 3’s lower reliability score compared to the other samples.

**4. The reported Cronbach's alpha values (0.75–0.85) indicate good internal consistency, but the manuscript could elaborate on how this compares to the original scale. Is there any trade-off in reliability due to the reduction in items?**

>> Thank you for this suggestion. We have added a sentence linking the internal consistency results from both scales (page 11, lines 195-198). Please note the instrument section of the paper also details these findings of the NIH-HEALS 35-item.

**5. It would also be helpful to discuss any potential limitations of using the short form, such as the potential loss of nuanced insights or sensitivity in detecting changes over time.**

>> We thank the reviewer for this invaluable insight. We added these potential limitations (page 13, lines 240-243).
